# Hypertrophic cardiomyopathy occurred after successful surgical correction of supravalvular aortic stenosis: a case report of Williams–Beuren syndrome

**DOI:** 10.3389/fped.2025.1580272

**Published:** 2025-08-08

**Authors:** Hongxiao Yu, Yang Ruan, Manfang Sun, Taole Li, Zhihua Nie

**Affiliations:** ^1^ Department of Cardiology, Haicang Hospital, Haicang, Xiamen, China; ^2^ The School of Clinical Medicine, Fujian Medical University, Fuzhou, Fujian, China; ^3^ Department of Neurology, Xiangya Hospital, Central South University, Changsha, Hunan, China

**Keywords:** Williams–Beuren syndrome, hypertrophic cardiomyopathy, supravalvular aortic stenosis, bioinformatics analysis, mavacamten

## Abstract

Williams–Beuren syndrome (WBS) is a multisystemic disorder caused by a microdeletion on chromosome 7q11.23.The supravalvular aortic stenosis (SVAS) is the most prevalent cardiovascular complication of WBS. However, hypertrophic cardiomyopathy (HCM) has rarely been reported in this population. We present a case of a patient with WBS who underwent successful surgical repair for SVAS in infancy but later developed HCM. Comprehensive genetic testing and further bioinformatic analysis revealed a deletion of approximately 1,486 kb at the 7q11.23 locus, and subsequent echocardiography demonstrated characteristic features of HCM. This case highlights the rare but clinically significant association between WBS and HCM, providing a foundation for further investigation into the biological mechanisms or potential biomarkers for HCM in WBS patients.

## 1 Background

Williams–Beuren syndrome (WBS) is a rare multisystemic genetic disorder resulting from a heterozygous deletion of approximately two megabases on chromosome 7q11.23. The global prevalence of WBS is estimated at 1 in 25,000 individuals from diverse populations (1). Characteristic features of WBS include a distinctive facial appearance, cardiovascular abnormalities, gastrointestinal dysfunction, and central nervous or endocrine disturbances (2). These phenotypes are primarily attributed to the deletion of several genes, such as *ELN*, *GTF2I*, *BAZ1B*, *LIMK1*, *STX1A*, and *MLXIPL* (3). Cardiovascular complications, particularly supravalvular aortic stenosis (SVAS), along with heart failure and arrhythmias, significantly worsen the clinical prognosis in patients with Williams–Beuren syndrome (WBS) (4). Hypertrophic cardiomyopathy (HCM), characterized by left ventricular hypertrophy, is the most common heritable cardiovascular disorder, with a prevalence of 1 in 500 in the general population (5). HCM is one of the most prevalent autosomal dominant inherited cardiac disorders, primarily caused by pathogenic mutations in genes encoding sarcomeric proteins. Since its initial genetic characterization, research has identified >1,400 distinct disease-causing mutations, the majority of which disrupt the function of either the thick myosin filaments within the cardiac sarcomere or the essential Z-disk components (6). Increasing evidence suggests that the phenotypic diversity of HCM may be influenced by modifying gene variants, epigenetic factors, and other regulatory mechanisms of gene expression (7). These findings underscore the need for further exploration into the complex genetic and molecular pathways that contribute to the variability in HCM phenotypes. Most patients with WBS exhibit myocardial hypertrophy secondary to aortic stenosis. However, the co-occurrence of WBS and HCM is rare, and the pathophysiological mechanisms underlying this association remain poorly understood. The relationship is likely multifactorial, involving intricate interactions between vascular and genetic factors. In this report, we present the case of a young male with WBS harboring a 1,486 kb deletion at the 7q11.23 locus. He subsequently developed HCM although he underwent successful surgical correction for aortic stenosis.

## 2 Case presentation

A 14-year-old male was referred to our hospital with complaints of dyspnea on exertion. The patient was found to have an ejection systolic murmur noted on physical examination at the age of 2.

**Figure 1:**
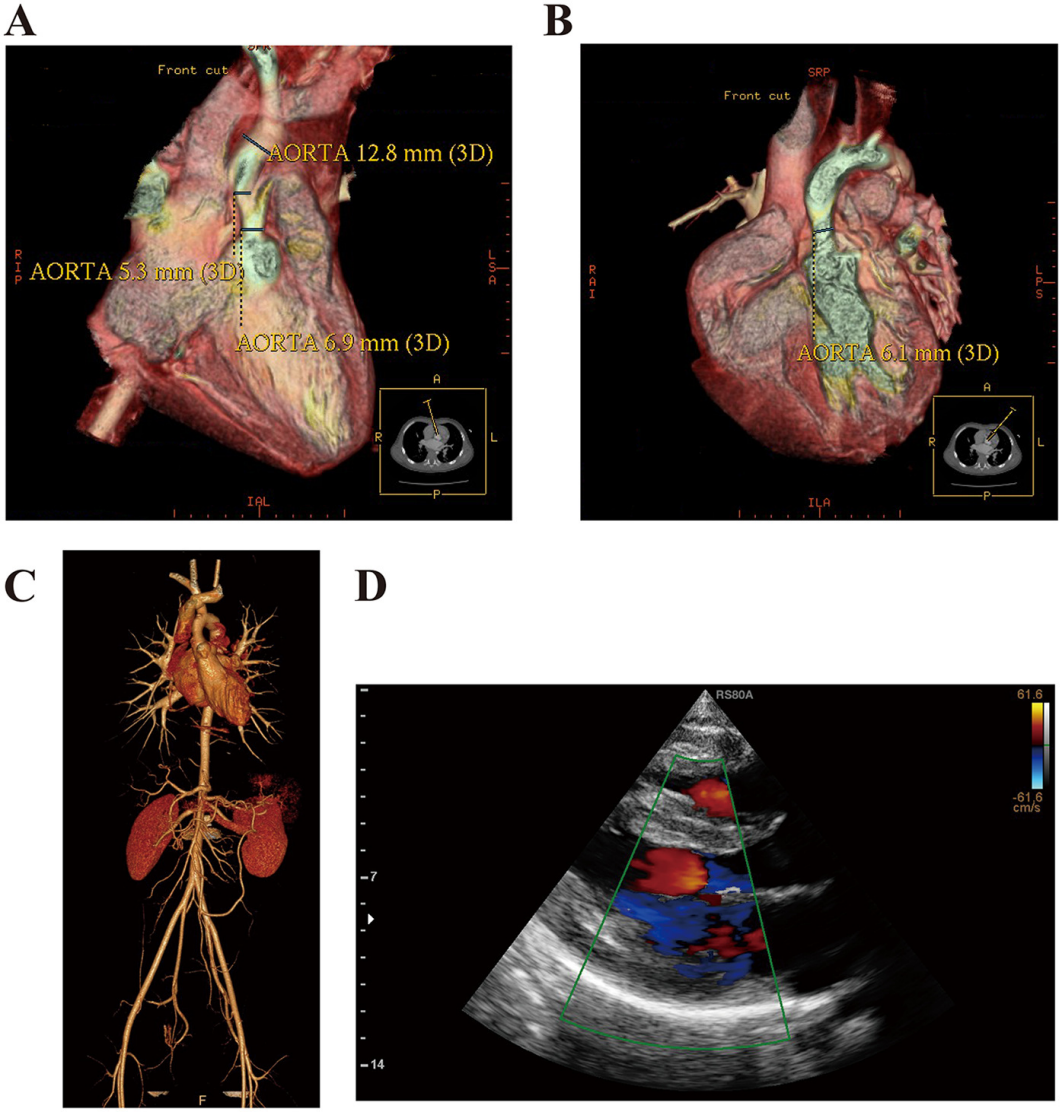
Case presentation and ancillary examination. **(A,B)** Thoracic aorta CTA confirmed the presence of SVAS in this patient at the age of 2 years. **(C)** Thoracic and abdominal aorta CTA confirmed that the aortic stenosis was cured after the surgery. **(D)** Echocardiography revealed the features of HCM. (IVS, interventricular septum; LVPW, left ventricular posterior wall; LVEDD, left ventricular end diastolic diameter; LA, left atrium; EF, ejection fraction).

The thoracic aorta computed tomography angiography (CTA) confirmed the presence of SVAS (Figures 1 A,B). Subsequently, the patient underwent surgical repair for the aortic stenosis. Postoperative echocardiography indicated a successful relief of the stenosis (Supplementary Figures A–D). Currently, the patient exhibits characteristic facial features, including ocular hypertelorism, a short neck, low-set ears, a broad nose with a depressed root, full lips, and mild hypertrichosis. Recently, he presented with new symptoms of chest distress and palpitation, which were particularly pronounced after activities, and a slight systolic murmur was detected at the left sternal border, between the second and third intercostal spaces.

## 3 Ancillary examination

We collected and reviewed the patient's demographic and clinical data. His heart rate and blood pressure were within normal limits (left arm blood pressure, 125/78 mmHg; right arm blood pressure, 130/75 mmHg; left leg blood pressure, 130/80 mmHg; right leg blood pressure, 131/70 mmHg). Laboratory results, including routine blood test, biochemical panel, blood glucose, and lipids, were all normal. Additionally, hormone levels for the thyroid, adrenal gland, and gonads were unremarkable. Tumor markers, including AFP, CEA, CA-199, and antinuclear and several rheumatoid antibodies, were all negative. The electrocardiography revealed the sinus rhythm. Neurological and cognitive assessments were also within normal limits. The CTA of the thoracic and abdominal aorta showed no recurrence of SVAS (Figure 1C). Echocardiography demonstrated the signs of HCM [interventricular septum (IVS), 15 mm; left ventricular posterior wall (LVPW), 11 mm; left ventricular end diastolic diameter (LVEDD), 45 mm; left atrium (LA), 39 mm; EF, 87%; aortic velocity, 1.0 m/s; left ventricular outflow tract pressure gradient, 15 mmHg; early maximal ventricular filling velocity, 0.6 m/s; atrial maximal ventricular filling velocity, 0.4 m/s] (Figure 1D) according to the establised guidelines (8).

## 4 WES-CNV

**Figure 2:**
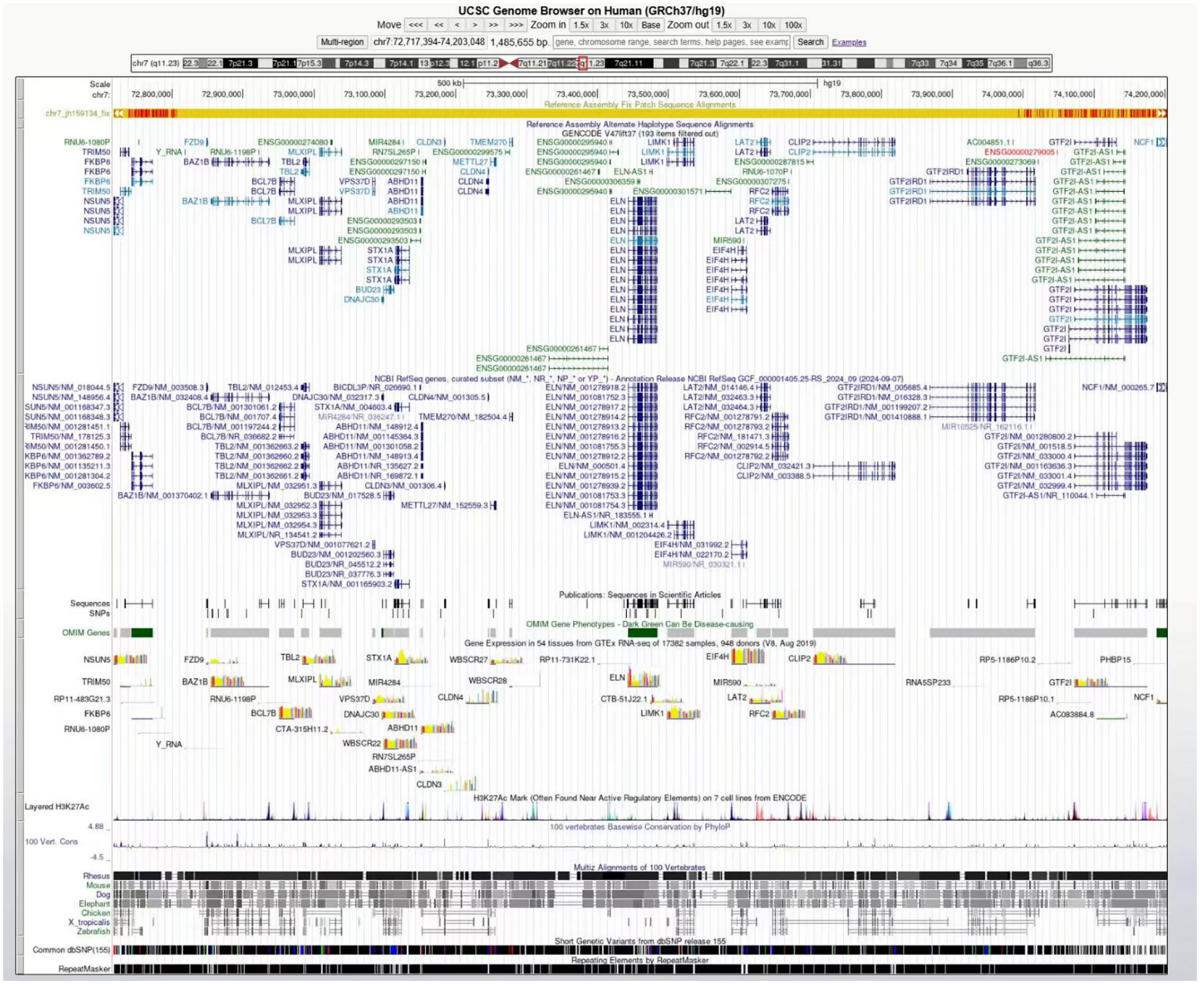
The genome browser (UCSC) described the deleted genes covered by the chromosome 7q11.23 region.

Blood samples were collected from the patient, and genomic DNA was extracted using the Magnetic Universal Genomic DNA kit (TIANGEN, China). The DNA was extracted, fragmented randomly, and purified using a magnetic particle-based method. Following this, the DNA fragments were ligated with adaptors and captured using the IDT xGen Exome Research Panel v1.0 (39M, Integrated DNA Technologies, USA) targeting the entire exome. The DNA libraries, after enrichment and purification, were sequenced on the SURFSeq 5000 sequencer according to the manufacturer's protocol (GeneMind, China). All sequencing reads were aligned to the reference human genome (UCSC hg19) by Burrows–Wheeler aligner (BWA). Local realignment and base quality recalibration were performed using GATK IndelRealigner and GATK BaseRecalibrator, respectively. Single-nucleotide variants (SNVs) or small indels were identified using GATK UnifiedGenotyper, and the variants were annotated with ANNOVAR. Then, the variants that were relevant to the patient's phenotype were selected and interpreted. Candidate variants were further validated in the probands and his relatives via PCR. The PCR products were subjected to direct sequencing on a 3500xL Genetic Analyzer (Applied Biosystems, USA) following the manufacturer's instructions. It showed a pathogenic variant with the chromosomal region of interest contains >26 protein-coding genes (*ABHD11*, *BAZ1B*, *BCL7B*, *BUD23*, *CLDN3*, *CLDN4*, *CLIP2*, *DNAJC30*, *ELN*, *EIF4H*, *FKBP6*, *FZD9*, *GTF2I*, *GTF2IRD1*, *LAT2*, *LIMK1*, *METTL27*, *MLXIPL*, *NCF1*, *NSUN5*, *RFC2*, *STX1A*, *TBL2*, *TMEM270*, *TRIM50*, *VPS37D*) (Figure 2). Moreover, no pathogenic variants were found in the primary HCM-related genes, specifically *MYH7*, *MYL2*, *MYL3*, *MYBPC3*, *TNNT2*, *TNNI3*, *TPM1*, and *ACTC1*. Among the family members of the patients, DNA analysis from the patient's mother, father, and sister revealed no similar mutations.

## 5 Diagnosis and treatment

Based on the results of clinical examination and WES-CNV, the patient was finally diagnosed with WBS with HCM. The patient initially showed a poor response to β-blockers but made significant progress after treatment with mavacamten, the first drug specifically designed for HCM, and it was approved following the publication of the EXPLORER-HCM trial (9). After 1 year of medication, the patient told us he did not feel chest distress or palpitation after activities.

## 6 Bioinformatic analysis

**Figure 3:**
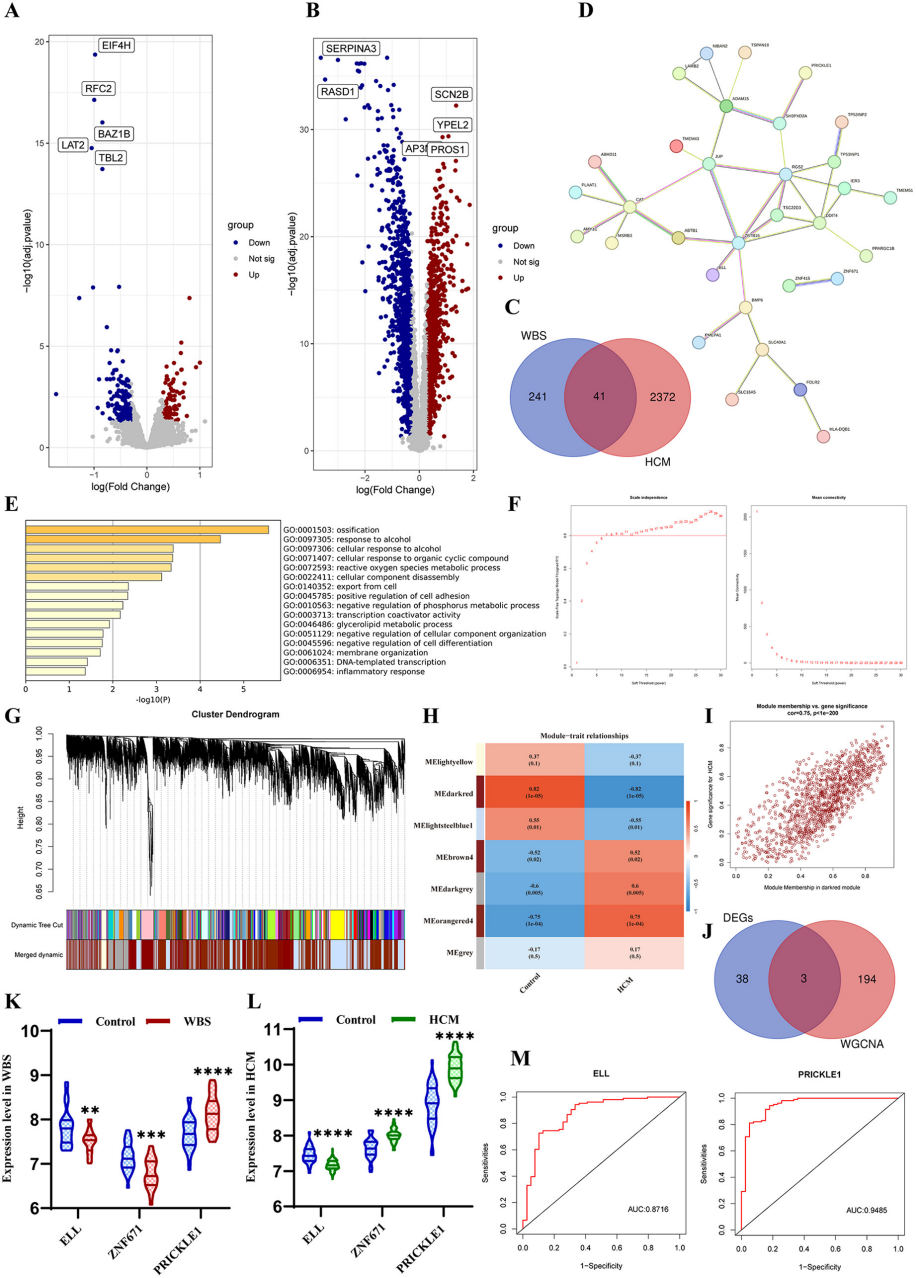
Bioinformatic analysis. **(A)** Volcano plot of DEGs between WBS vs. control. **(B)** Volcano plot of DEGs between HCM vs. control. **(C)** Venn diagram of DEGs for both diseases. **(D)** PPI networks of common DEGs. **(E)** Biological enrichment analysis of DEGs. **(F)** Estimation of the scale independence and mean connectivity for soft thresholding value. **(G)** Cluster dendrogram module division and merge. **(H)** Correlation between modules and phenotypes of HCM. **(I)** Scatterplot of GS vs. MM in the darkred. **(J)** Venn diagram of the hub genes between DEGs and WGCNA. **(K)** The expression level of three hub genes in WBS patients. **(L)** The expression level of three hub genes in HCM patients. **(M)** ROC curves indicated the predictive value of ELL and PRICKLE1 in HCM.

RNA sequencing data for WBS and HCM patients were obtained from Gene Expression Omnibus (GEO, https://www.ncbi.nlm.nih.gov/geo). The datasets processing included background correction, log2 transformation, and normalization using the “affy” and “geoquery” R packages, followed by batch effects removal with the “sva” package. Differentially expressed genes (DEGs) were identified by using the R package “limma.” The adjusted *p*-value < 0.05 and the log2|FC| > 0.3 were considered to be the statistical criteria. Based on the GSE89594, we identified 282 DEGs significantly differentially expressed in 32 WBS patients compared with 30 control samples (Figure 3A). Based on the GSE36961, 2,413 DEGs were identified in 106 HCM patients compared with 39 control samples (Figure 3B). We obtained a total of 41 DEGs with the intersection of both datasets (Figure 3C), and their interaction was visualized (Figure 3D). Gene Ontology (GO) and Kyoto Encyclopedia of Genes and Genomes (KEGG) enrichment analyses showed that the common DEGs were significantly involved in ossification, cellular response to alcohol and organic cyclic compound, reactive oxygen species metabolic process, and cellular component disassembly (Figure 3E). Additionally, Weighted Gene Co-expression Network Analysis (WGCNA) was applied using the R package “WGCNA” based on the GSE36961. The soft thresholding power of 7 was selected to construct a scale-free co-expression network with an independence degree of ≥0.8 (Figure 3F). The DEGs were then clustered to detect the hub modules, and seven key modules, with a similar trait profile, were identified based on MEDiss Thres = 0.4 (Figure 3G). The correlation between harvested modules was calculated, and the darkred module (Cor = −0.82, *p* = 1 × 10^−5^) was significantly associated with HCM (Figure 3H). With the cutoff criteria of gene significance (|GS| ≥ 0.6) and module membership (|MM| ≥ 0.7), we obtained 197 genes most strongly related to HCM (Figure 3I). Finally, three hub genes were identified from the intersection between DEGs and WGCNA (Figure 3J). Among these, *ELL* was consistently downregulated in both WBS and HCM patients, while *PRICKLE1* was consistently upregulated (Figures 3K,L). Finally, the receiver operating characteristic (ROC) curve analysis also demonstrated that both *ELL* and *PRICKLE1* had strong diagnostic potential for HCM (Figure 3M), suggesting both genes may serve as the signature biomarkers for HCM secondary to WBS.

## 7 Discussion

This case report describes a rare presentation of WBS in a pediatric patient complicated by HCM. The patient was initially diagnosed with SVAS at 2 years of age and subsequently underwent successful surgical repair involving patch aortaplasty. The management of cardiovascular manifestations in WBS requires careful consideration, with therapeutic options spanning from conservative approaches to surgical interventions. Among these, SVAS correction represents the most common indication for surgical treatment, particularly given its association with significantly reduced life expectancy (averaging only 35 years) when left untreated (10). For pediatric patients with SVAS, particularly complicated by chronic cardiac insufficiency, the aortic reconstruction is recommended. Early surgical intervention is critical for preventing adverse outcomes, such as ventricular hypertrophy, heart failure, and sudden cardiac death (11). The symptoms of this patient were significantly alleviated postoperatively, and follow-up echocardiography and CTA showed no recurrence of the stenosis. However, persistent progressive myocardial thickening in this patient occurred despite effective relief of the aorta stenosis. Twelve years after the surgery, echocardiography indicated the presence of ventricular hypertrophy, manifesting as asymmetric septal hypertrophy (IVS/LVPW = 1.363), while showing normal diastolic function (E/A ratio = 1.5) and absence of outflow tract obstruction (left ventricular outflow tract pressure gradient = 15 mmHg). In the WBS, myocardial hypertrophy most commonly occurs as a secondary manifestation of aortic stenosis. This afterload-induced cardiac adaptation characteristically manifests as concentric, symmetric left ventricular hypertrophy. A notable clinical feature accompanying this pathophysiology is a significant blood pressure gradient between the upper and lower extremities. In contrast, familial HCM, predominantly caused by genetic mutations, most commonly presents with asymmetric septal hypertrophy and may be complicated by left ventricular outflow tract obstruction (12). This patient exhibited no evidence of significant myocardial hypertrophy during the surgical intervention at two years of age. It suggests the observed ventricular hypertrophy is not a simple cardiac hypertrophy due to the prior SVAS, but rather to a primary HCM with genetic predisposition secondary to WBS.

The genetic mechanisms underlying the co-occurrence of WBS and HCM remain unreported in the literature. Current evidence suggests this association may be attributable to microdeletions at the 7q11.23 locus, which encompasses multiple genes. Among these, the ELN gene appears most strongly associated with cardiovascular pathology. ELN encodes elastin, the primary structural component of elastic fibers in human connective tissue that provides critical elasticity to large arteries. Insufficiency of ELN disrupts normal vascular architecture, leading to characteristic pathological changes including vessel wall thickening, loss of elastic properties, and subsequent luminal stenosis (13). As demonstrated in our case report, deletion of the ELN gene is the direct etiological factor for SVAS. Previous studies have confirmed that ELN gene deletion is specifically associated with SVAS and connective tissue abnormalities in WBS patients, while showing no correlation with other clinical manifestations (3). In this case, the patient's normal neurologic examination and endocrine profile further underscore the important role of the ELN gene in this pathological process. Notably, there is no evidence for a direct association between ELN gene mutations and HCM. It has been shown that ELN gene deletion induced cardiac hypertrophy in a mouse model may be related to NOX-mediated oxidative stress (14). However, other research indicated a positive correlation between ELN gene upregulation and cardiac hypertrophy in mice (15). These contradictory findings suggest the relationship between ELN gene expression and HCM needs to be further investigated.

Furthermore, based on the GEO databases and through bioinformatics analysis, we identified two genes, the *ELL* gene and *PRICKLE1* gene, that may serve as potential hub genes in WBS complicated by HCM. The *ELL* gene encodes eleven-nineteen lysine-rich leukemia protein, part of the super elongation complex, which plays a crucial role in RNA transcription initiation by promoting the stabilization and recruitment of RNA polymerase II (16). It has been reported that *ELL*-associated factor 2 (Eaf2) knockout can not only induce tumorigenesis in multiple tissues but also exacerbate cardiac hypertrophy in a mouse model (17). *PRICKLE1* is a key component of the planar cell polarity (PCP) pathway, which participates in the establishment of cell polarity during embryonic development (18). *PRICKLE1* was identified as the target gene of MESP1, which marks the cardiovascular progenitors and promotes cardiovascular differentiation (19). Bioinformatics results suggested that downregulation of *ELL* or upregulation of *PRICKLE1* may play a crucial role in the WBS with HCM. Although these two genes may not directly cause HCM, changes in their encoded protein expression levels could serve as potential biomarkers for WBS-associated HCM, and related signal transduction pathways may contribute to the occurrence and development of primary HCM in WBS. Nevertheless, these are only initial hypotheses; sample cohorts and experimental validation at the cellular and molecular level are essential.

Mavacamten, a selective and allosteric small-molecule cardiac myosin inhibitor, has shown promising results in treating HCM. It can reduce the myocardial hyperactivity or hypercontractility, especially alleviating the left ventricular filling pressure and outflow tract obstruction (20). Additionally, the mavacamten has been demonstrated to reduce the Ca2^+^ sensitivity and myocardial energy demand, thus relieving the diastolic dysfunction (21). Given the absence of left ventricular outflow tract obstruction, the patient demonstrated obvious improvement in symptoms of the cardiovascular system following 1 year of mavacamten intervention. Clinical studies have shown significant improvements in the status of both non-obstructive HCM (nHCM) and obstructive HCM (oHCM) patients treated with mavacamten, regardless of concurrent use of β-blockers (22). A MAVERICK-HCM clinical trial (Mavacamten in Adults with Symptomatic Non-obstructive Hypertrophic Cardiomyopathy) also demonstrated the safety and efficacy of mavacamten in most subjects with symptomatic nHCM (23). This highlights the therapeutic potential of mavacamten as a targeted treatment for broad-spectrum HCM, including cases arising from particular syndromes such as WBS.

While our study provides novel insights, several limitations should be addressed in future research. The bioinformatic analysis results represent preliminary scientific hypotheses that require validation through *in vitro* and *in vivo* experiments. Subsequent investigations should focus on establishing larger WBS cohort studies to compare *ELL* and *PRICKLE1* expression levels with healthy populations and collecting more comprehensive genomic data to elucidate the molecular mechanisms underlying HCM pathogenesis. Despite these limitations, our work offers a valuable framework for investigating biological mechanisms in WBS-associated HCM and may contribute to the identification of potential genetic therapeutic targets for affected patients.

## Data Availability

Publicly available datasets were analyzed in this study. This data can be found in the GEO database (http://www.ncbi.nlm.nih.gov/geo), accession numbers: GSE89594 and GSE36961.
