# Supplementary material for: Hypertrophic cardiomyopathy occurred after successful surgical correction of supravalvular aortic stenosis: a case report of Williams–Beuren syndrome
Source: Front Pediatr. 2025 Aug 8;13:1580272. doi: 10.3389/fped.2025.1580272 (PMC12370502; doi:10.3389/fped.2025.1580272)
Supplement: Supplementary file 1 [file Supplementaryfile1.docx]

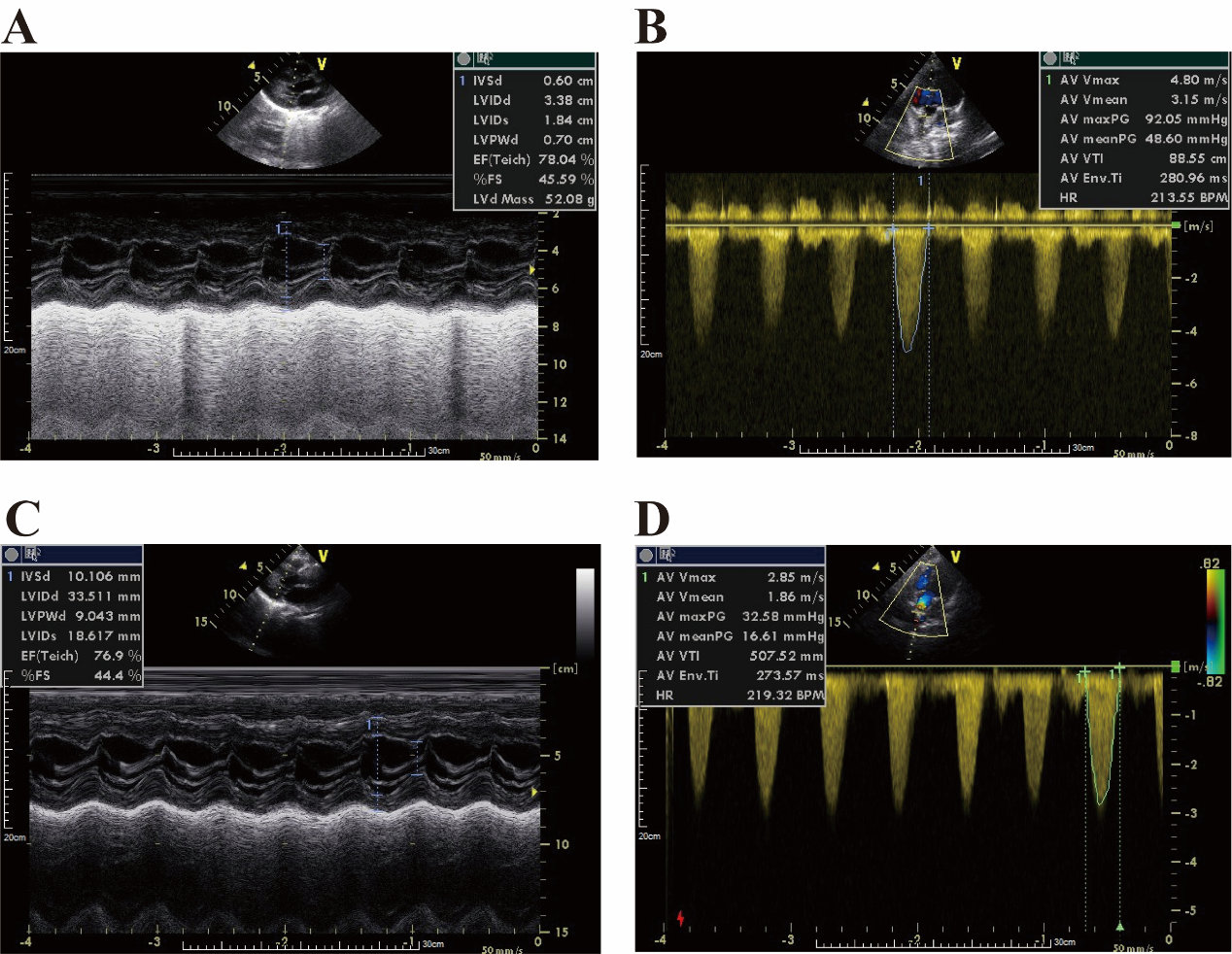


**Supplementary Fig**. Echocardiography of the patient before and after the surgery for supravalvular aortic stenosis. (A,B) Echocardiographic of the patient at 2 years of age (before surgery) (IVS: 6mm, LVPW: 7mm, Aortic velocity: 4.8m/s, Left ventricular outflow tract pressure gradient: 49mmHg). (C,D) Echocardiographic of the patient at 4 years of age (2 years after surgery) (IVS: 10mm, LVPW: 9mm, Aortic velocity: 2.8m/s, Left ventricular outflow tract pressure gradient: 17mmHg).
